# Are fewer cases of diabetes mellitus diagnosed in the months after SARS-CoV-2 infection? A population-level view in the EHR-based RECOVER program

**DOI:** 10.1017/cts.2023.34

**Published:** 2023-03-08

**Authors:** Neha V. Reddy, Hsin-Chieh Yeh, Jena S. Tronieri, Til Stürmer, John B. Buse, Jane E. Reusch, Steven G. Johnson, Rachel Wong, Richard Moffitt, Kenneth J. Wilkins, Jeremy Harper, Carolyn T. Bramante, the N3C and RECOVER Consortiums

**Affiliations:** 1 Division of General Internal Medicine, Department of Medicine, University of Minnesota Medical School, Minneapolis, MN, USA; 2 Departments of Medicine, Epidemiology and Oncology, Welch Center for Prevention, Epidemiology, and Clinical Research, Johns Hopkins University, Baltimore, MD, USA; 3 Department of Psychiatry, Perelman School of Medicine at the University of Pennsylvania, Philadelphia, PA, USA; 4 Department of Epidemiology, Gillings School of Global Public Health, University of North Carolina at Chapel Hill, Chapel Hill, NC, USA; 5 Division of Endocrinology, Department of Medicine, University of North Carolina Medical School, Chapel Hill, NC, USA; 6 University of Colorado Denver Anschutz Medical Campus, Denver, CO, USA; 7 Institute for Health Informatics, University of Minnesota, Minneapolis, MN, USA; 8 Department of Biomedical Informatics, Stony Brook University, Stony Brook, NY, USA; 9 Biostatistics Program, Office of the Director, National Institute of Diabetes and Digestive and Kidney Disease, Bethesda, MD, USA; 10 Owl HealthWorks, Indianapolis, IN, USA

**Keywords:** COVID-19, epidemiology, PASC, type 2 diabetes, new diabetes

## Abstract

Long-term sequelae of severe acute respiratory coronavirus-2 (SARS-CoV-2) infection may include increased incidence of diabetes. Here we describe the temporal relationship between new type 2 diabetes and SARS-CoV-2 infection in a nationwide database. We found that while the proportion of newly diagnosed type 2 diabetes increased during the acute period of SARS-CoV-2 infection, the mean proportion of new diabetes cases in the 6 months post-infection was about 83% lower than the 6 months preinfection. These results underscore the need for further investigation to understand the timing of new diabetes after COVID-19, etiology, screening, and treatment strategies.

## Introduction

The relationship between severe acute respiratory coronavirus 2 (SARS-CoV-2), the COVID-19 pandemic, and diabetes mellitus (DM) is the subject of active investigation [1,2]. A growing body of evidence suggests a possible increased incidence of new-onset DM after infection with SARS-CoV-2 [3–5]. The mechanism by which SARS-CoV-2 infection increases the risk of developing DM is not fully understood. Hypotheses include stress hyperglycemia related to acute illness, direct effect of the virus on pancreatic vasculature or beta cells, changes in innate immunity, and iatrogenic causes [6–8].

However, risk factors for developing diabetes likely increased for the entire population, regardless of infection with SARS-CoV-2, as rates of overweight and obesity increased at the population level [9–11]. In addition, physical activity declined globally during the pandemic and has not recovered [12]. Stress related to the pandemic and subsequent increased endogenous cortisol may also be a risk factor for DM independent of SARS-CoV-2 infection [10,13,14]. It is currently unknown if rates of incident DM are higher after infection with SARS-CoV-2 than after infection with similar non-COVID-19 viruses [3–5].

We sought to characterize the temporal relationship between new type 2 diabetes diagnoses relative to SARS-CoV-2 infection in individuals who had SARS-CoV-2 and were also diagnosed with type 2 DM within 6 months of the start of the pandemic. We hypothesized that there would be an increase in new diagnoses of type 2 DM after infection with SARS-CoV-2. We undertook this analysis in a large, nationally representative database in the USA, the National Covid Cohort Collaborative (N3C).

## Methods

This is a nationwide cross-sectional analysis to display the temporal relationship between new onset type 2 diabetes relative to SARS-CoV-2 infection in the N3C database. The N3C database aggregates data from electronic health records (EHR) from more than 70 institutions nationally to accelerate research efforts on the evolving COVID-19 pandemic [15].

The study sample included all persons with SARS-CoV-2 and with type 2 DM, from March 2020 to February 2022 in the N3C Database. To reduce ascertainment and selection bias, the sample was restricted to patients who had at least one outpatient clinical encounter at least six months prior to their SARS-CoV-2 infection, and at least 6 months of follow-up data. Because the earliest data in the N3C database are from 2018, everyone in the sample had engaged with the healthcare system at least once between 6 and 21 months prior to their SARS-CoV-2 infection.

We defined the index date, day 1, as the date of first SARS-CoV-2 infection by either positive SARS-CoV-2 PCR test or ICD-10 diagnosis code.

Type 2 diabetes was defined by ICD code using ICD lists reviewed for completeness and accuracy by two clinicians [16]. New type 2 DM was defined as the earliest problem list code for type 2 DM, in persons who did not have an ICD code for type 2 DM in the EHR before September 2019. Because the sample was restricted to persons who had at least 6 months of EHR data before an infection with SARS-CoV-2, the earliest DM diagnoses in the sample would be September 2019. All problem list items in N3C are mapped to SNOMED, and 523 SNOMED codes were identified by clinicians as a diagnosis of type 2 DM (full list included in supplement). The earliest DM diagnosis date was subtracted from the index date per subject to bucket them by 30-day time periods relative to the index date. The bucket including the date of SARS-CoV-2 infection included new type 2 DM diagnoses 7 days prior to the index date to account for SARS-CoV-2 PCR tests that resulted after the initiation of a clinical encounter for suspected COVID-19. The first analysis evaluated the proportion of type 2 DM diagnoses in the N3C database that were new diagnoses (first diagnosis in someone who previously did not have a diagnosis of diabetes). This included taking the average proportion of new diabetes cases in the 6 months prior to SARS-CoV-2 and the 6 months after, both including and excluding the 30 days immediately before and after infection. When calculating the mean proportions of new diabetes cases from 6 months before and after infections, we used only the 6 months after infection for this comparison because only a subset of those newly diagnosed with diabetes had follow-up longer than 6 months post-infection. The second analysis examined the raw number of new type 2 DM diagnoses. Both analyses are presented by calendar month of the pandemic. Analyses were conducted in the secure computing environment using Palantir and R statistical software (R Foundation for Statistical Computing, Vienna, Austria).

## Results

**Fig. 1.**
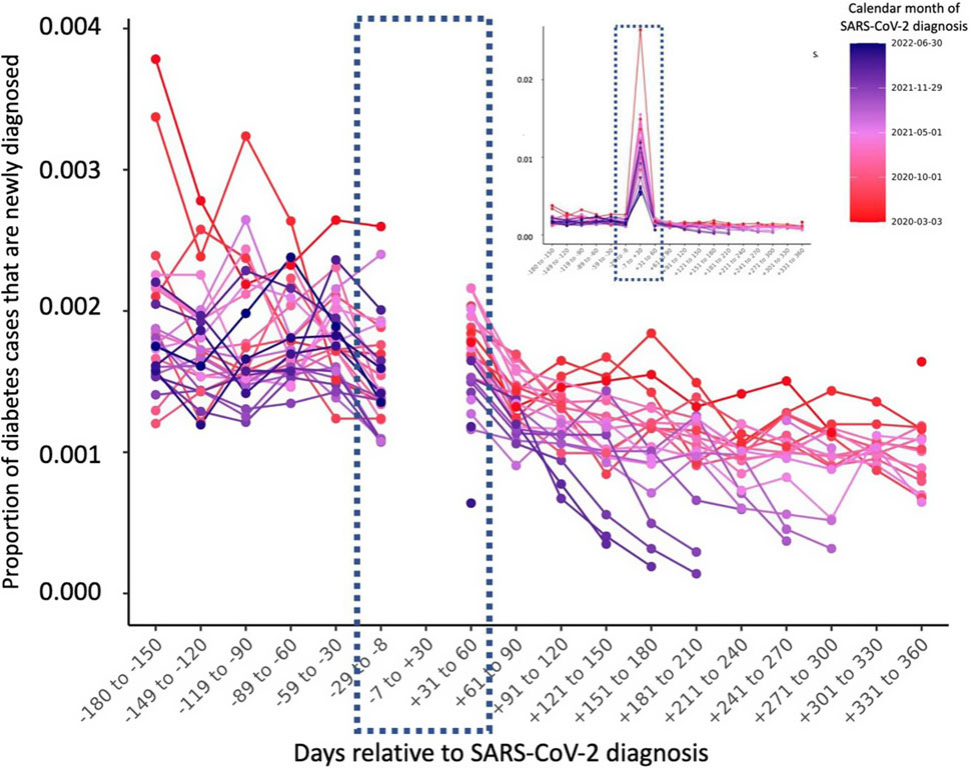
Among all persons with diabetes in the database, the proportion of diabetes cases that were newly diagnosed between September 2019 and February 2022, by 30-day periods relative to infection with SARS-CoV-2. This plot represents the proportion of diabetes cases in the N3C database that were diagnosed after September 2019 in persons who did not have a previous diagnosis of diabetes. Each line represents a calendar month of the pandemic. The Y-axis is the proportion of all persons with SARS-CoV-2 and a DM diagnosis who received the ICD code for DM after September 2019 and the temporal relationship between the diagnoses between 180 days prior to 360 days after the SARS-CoV-2 infection. The large peak between 8 days before and 30 days after is represented in an inset so that the top of that peak is visible without compressing the Y-axis.

**Fig. 2.**
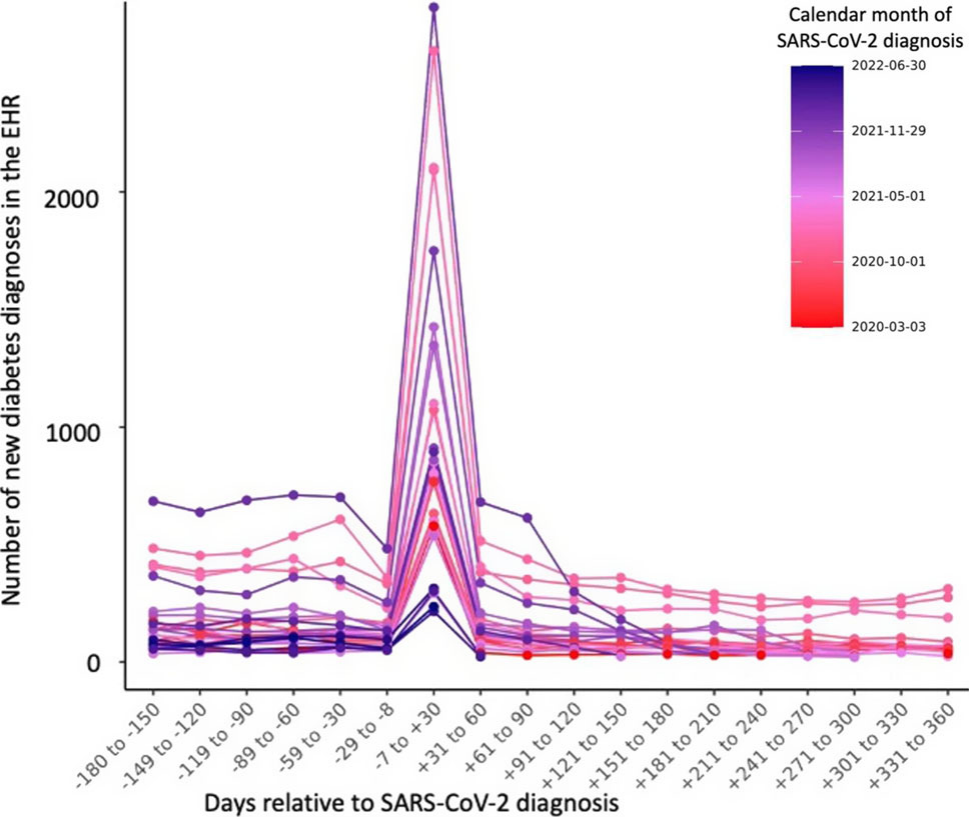
Number of new diabetes mellitus (DM) cases within 180 before and 360 days after SARS-CoV-2 infection, among individuals who have both EHR-recorded SARS-CoV-2 infection and ICD code for DM and did not have an ICD code for DM before September 2019. Each line represents a calendar month during the pandemic; the Y-axis is the number of new DM cases in the N3C database in persons with a documented SARS-CoV-2 infection. The X-axis is temporal relationship between the diabetes diagnosis relative to each individual’s SARS-CoV-2 infection.

Figures 1 and 2 present the temporal relationship between SARS-CoV-2 infection and new diagnoses of DM. Figure 1 shows the proportion of type 2 diabetes diagnoses that are new diagnoses, by 30-day windows relative to the index date of SARS-CoV-2 infection. Figure 2 shows the number of new DM cases in the N3C, by 30-day windows relative to SARS-CoV-2 infection. DM diagnosed in the 7 days prior to SARS-CoV-2 infection could indicate that the DM and SARS-CoV-2 were diagnosed in the same healthcare encounter, due to lag times in SARS-CoV-2 results.

The mean proportion of new diabetes cases in the 6 months after infection (day −7 to day 180) is about 83% lower than the mean proportion of new diabetes cases in the 6 months before infections (day −180 to day −8) when including the 30 days immediately pre- and post-infection (Figure 1). This trend continues when excluding the acute 30 days before and 30 days after SARS-CoV-2: the average proportion of new diabetes cases from day −180 to −30 is 0.19 and the average proportion of new cases from day +31 to +180 is 0.13, suggesting that the proportion of new diabetes cases after SARS-CoV-2 infection is about 43% lower than before when excluding the acute increase in new diabetes right around the time of infection.

The count of new DM diagnoses is lower in the months after SARS-CoV-2 infection than in the months before (Figure 2). Supplementary Tables 1 and 2 contain the data that were used to make the figures and calculate the averages. Demographic characteristics of all persons with SARS-CoV-2 infection in the N3C database are presented in Supplementary Table 3.

## Discussion

This is a cross-sectional, population-level assessment of the temporal relationship between SARS-CoV-2 infection and new diagnoses of type 2 DM persons who have both conditions in the N3C database. We observed a sharp increase in new cases of type 2 DM within 7 days before to 30 days after SARS-CoV-2 infection, followed by a decrease in new DM diagnoses. It also appears that the number and proportion of new-onset DM may then be lower in the months after SARS-CoV-2 infection than in the months preceding.

It is possible that the sharp peak in type 2 DM cases represents a marked increase of interaction with the healthcare system at the time of SARS-CoV-2 infection. Hemoglobin A1C is the most commonly used criteria for diagnosing diabetes. Because of the general requirement for confirmation of lab criteria for the diagnosis of diabetes and the asynchronous documentation of ICD codes at an encounter with the return of lab results, clinicians may not enter an ICD code for DM after just one episode of hyperglycemia or two episodes of hyperglycemia in the same healthcare encounter. Thus, the spike of new type 2 DM cases immediately surrounding SARS-CoV-2 infections could represent individuals who were newly interacting with the healthcare system, having their hemoglobin A1C or glucose value tested, and receiving a diagnosis of DM based on the lab result.

A recent retrospective cohort study reported an increased risk of incident DM during the post-acute infection period in the US Department of Veterans Affairs (VA) database [5]. The VA population has guaranteed access to healthcare, whereas the spike followed by a decrease in new-onset DM in the N3C population may reflect patients newly interacting with the healthcare system who are simply *diagnosed* with DM that they already had because of this interaction with healthcare. The difference between the N3C results, acute increases in new diabetes, and the VA data, which demonstrate a continuous increase in incident diabetes, could be related to periodic (VA) rather than acute (N3C) interaction with the medical system. Overall, it will be important to follow the population burden of new diabetes post-pandemic to disentangle the relationship to SARS-CoV-2 exposure or other environmental factors.

Another potential explanation for a large peak in type 2 DM cases is that the physiologic stress of infection with SARS-CoV-2 does push high-risk individuals to develop DM. The decrease in new DM diagnoses in the months after SARS-CoV-2 infection could reflect the metabolic challenge of the infection revealing DM that would have presented later in the absence of SARS-CoV-2 infection. As such, the SARS-CoV-2 infection caused individuals to develop diabetes earlier, or be diagnosed earlier, thereby decreasing the population at risk for DM in the months after SARS-CoV-2 infection.

Understanding the population at risk for diabetes is difficult when considering the effects of the pandemic on the population as a whole – increased stress, weight, central adiposity, and decreased physical activity. Thus, the risk of developing diabetes has potentially increased regardless of infection with SARS-CoV-2. It is also possible that infection with SARS-CoV-2 does precipitate new diagnoses of DM in persons who would not have developed diabetes otherwise, and that with longer follow-up the slope of new DM diagnoses will go up in persons who have been infected with SARS-CoV-2.

This is not a causal analysis and should not be interpreted as such. Our analysis suggests that there is a spike in type 2 DM diagnoses immediately surrounding SARS-CoV-2 infection, followed by a decrease in new diagnoses. Such a pattern may be attributable to increased interaction with the healthcare system, misclassification of prevalent disease as incident, or the stress of the SARS-CoV-2 infection [18]. Rigorous epidemiologic and mechanistic studies are needed to understand whether there are causal relationships between SARS-CoV-2 infection and the development of type 2 DM in the short- and long term. Clinical trial cohorts may represent an opportunity for prospective, complete data for assessing the prevalence of new-onset type 2 DM in those infected with SARS-CoV-2. The main conclusion from this cross-sectional analysis of the temporal relationship between SARS-CoV-2 infection and type 2 DM is that the interplay between COVID-19, pandemic-related lifestyle chances, and DM is complex and must be studied carefully with an appropriate control cohort.

## Supplementary material

For supplementary material accompanying this paper visit https://doi.org/10.1017/cts.2023.34. click here to view supplementary material
