# Supplementary material for: Are fewer cases of diabetes mellitus diagnosed in the months after SARS-CoV-2 infection? A population-level view in the EHR-based RECOVER program
Source: J Clin Transl Sci. 2023 Mar 8;7(1):e90. doi: 10.1017/cts.2023.34 (PMC10130848; doi:10.1017/cts.2023.34)
Supplement: Supplementary file 1 [file S2059866123000341sup001.docx]

**Supplementary Appendix**

**Supplementary Table 1: Proportion of individuals in the electronic health record database with type 2 diabetes mellitus whose diabetes was diagnosed after September 2019. These data are also represented in for Figure 1.**

| Month of  Infection | -180  to  -150 | -149  to  -120 | -119  to  -90 | -89  to  -60 | -59  to  -30 | -29  to  -8 | -7  to  +30 | 31  to  60 | 61  to  90 | 91  to  120 | 121  to  150 | 151  to  180 | 180  to  210 | 211  to  240 | 241  to  270 | 271  to  300 | 301  to  330 | 331  to  360 |
| --- | --- | --- | --- | --- | --- | --- | --- | --- | --- | --- | --- | --- | --- | --- | --- | --- | --- | --- |
| Mean | 0.00202 | 0.00182 | 0.00186 | 0.00188 | 0.00184 | 0.00154 | 0.01227 | 0.00172 | 0.00128 | 0.00118 | 0.00112 | 0.00102 | 0.00109 | 0.00095 | 0.00103 | 0.00101 | 0.00109 | 0.00104 |
| Mar-20 | 0.0038 | 0.0028 | 0.0022 | 0.0024 | 0.0027 | 0.0025 | 0.0263 | 0.0017 | 0.0013 | 0.0015 | 0.0015 | 0.0015 | 0.0013 | 0.0014 | 0.0015 | 0.0011 |  | 0.0017 |
| Apr-20 | 0.0038 | 0.0026 | 0.0026 | 0.0033 | 0.0027 | 0.0018 | 0.0225 | 0.0018 | 0.0015 | 0.0012 | 0.0014 | 0.0016 | 0.0014 | 0.0013 | 0.0011 | 0.0014 | 0.0013 | 0.0013 |
| May-20 | 0.0033 | 0.0024 | 0.0033 | 0.0026 | 0.0016 | 0.0014 | 0.0153 | 0.0019 | 0.0014 | 0.0015 | 0.0016 | 0.0019 | 0.0015 | 0.0011 | 0.0013 | 0.0014 | 0.0014 | 0.0012 |
| Jun-20 | 0.0021 | 0.0026 | 0.0025 | 0.0019 | 0.0017 | 0.0018 | 0.0141 | 0.0020 | 0.0013 | 0.0016 | 0.0017 | 0.0014 | 0.0010 | 0.0012 | 0.0011 | 0.0012 | 0.0013 | 0.0012 |
| Jul-20 | 0.0024 | 0.0018 | 0.0016 | 0.0018 | 0.0013 | 0.0013 | 0.0115 | 0.0017 | 0.0014 | 0.0014 | 0.0008 | 0.0011 | 0.0012 | 0.0011 | 0.0012 | 0.0011 | 0.0009 | 0.0007 |
| Aug-20 | 0.0019 | 0.0012 | 0.0018 | 0.0018 | 0.0021 | 0.0019 | 0.0124 | 0.0019 | 0.0017 | 0.0013 | 0.0010 | 0.0012 | 0.0014 | 0.0012 | 0.0011 | 0.0009 | 0.0010 | 0.0011 |
| Sep-20 | 0.0012 | 0.0014 | 0.0016 | 0.0023 | 0.0017 | 0.0018 | 0.0110 | 0.0018 | 0.0013 | 0.0010 | 0.0010 | 0.0012 | 0.0009 | 0.0010 | 0.0013 | 0.0011 | 0.0011 | 0.0009 |
| Oct-20 | 0.0013 | 0.0017 | 0.0015 | 0.0016 | 0.0017 | 0.0015 | 0.0099 | 0.0017 | 0.0012 | 0.0014 | 0.0012 | 0.0013 | 0.0012 | 0.0010 | 0.0011 | 0.0009 | 0.0010 | 0.0008 |
| Nov-20 | 0.0017 | 0.0015 | 0.0016 | 0.0015 | 0.0017 | 0.0013 | 0.0084 | 0.0016 | 0.0014 | 0.0013 | 0.0012 | 0.0012 | 0.0010 | 0.0009 | 0.0010 | 0.0010 | 0.0010 | 0.0011 |
| Dec-20 | 0.0018 | 0.0017 | 0.0018 | 0.0020 | 0.0024 | 0.0014 | 0.0099 | 0.0020 | 0.0017 | 0.0013 | 0.0013 | 0.0012 | 0.0011 | 0.0010 | 0.0010 | 0.0010 | 0.0010 | 0.0012 |
| Jan-21 | 0.0021 | 0.0020 | 0.0022 | 0.0024 | 0.0018 | 0.0012 | 0.0113 | 0.0022 | 0.0015 | 0.0013 | 0.0011 | 0.0012 | 0.0012 | 0.0010 | 0.0010 | 0.0012 | 0.0011 | 0.0010 |
| Feb-21 | 0.0022 | 0.0020 | 0.0024 | 0.0016 | 0.0019 | 0.0015 | 0.0147 | 0.0022 | 0.0016 | 0.0015 | 0.0015 | 0.0013 | 0.0011 | 0.0008 | 0.0010 | 0.0012 | 0.0010 | 0.0009 |
| Mar-21 | 0.0022 | 0.0023 | 0.0016 | 0.0015 | 0.0021 | 0.0019 | 0.0138 | 0.0019 | 0.0016 | 0.0012 | 0.0011 | 0.0010 | 0.0009 | 0.0012 | 0.0011 | 0.0010 | 0.0009 | 0.0007 |
| Apr-21 | 0.0018 | 0.0016 | 0.0016 | 0.0015 | 0.0022 | 0.0016 | 0.0123 | 0.0017 | 0.0012 | 0.0012 | 0.0010 | 0.0009 | 0.0010 | 0.0007 | 0.0008 | 0.0006 | 0.0011 | 0.0011 |
| May-21 | 0.0017 | 0.0018 | 0.0021 | 0.0021 | 0.0018 | 0.0018 | 0.0152 | 0.0020 | 0.0014 | 0.0013 | 0.0012 | 0.0010 | 0.0012 | 0.0011 | 0.0010 | 0.0009 | 0.0011 | 0.0007 |
| Jun-21 | 0.0018 | 0.0020 | 0.0024 | 0.0018 | 0.0021 | 0.0024 | 0.0142 | 0.0015 |  |  | 0.0015 |  | 0.0012 |  | 0.0013 | 0.0012 |  |  |
| Jul-21 | 0.0020 | 0.0017 | 0.0015 | 0.0019 | 0.0017 | 0.0011 | 0.0122 | 0.0013 | 0.0010 | 0.0013 | 0.0009 | 0.0006 | 0.0010 | 0.0006 | 0.0007 | 0.0006 |  |  |
| Aug-21 | 0.0016 | 0.0017 | 0.0014 | 0.0016 | 0.0013 | 0.0011 | 0.0105 | 0.0011 | 0.0010 | 0.0011 | 0.0010 | 0.0009 | 0.0010 | 0.0010 | 0.0005 | 0.0003 |  |  |
| Sep-21 | 0.0016 | 0.0016 | 0.0015 | 0.0016 | 0.0016 | 0.0011 | 0.0109 | 0.0016 | 0.0013 | 0.0011 | 0.0010 | 0.0010 | 0.0013 | 0.0007 | 0.0004 |  |  |  |
| Oct-21 | 0.0018 | 0.0014 | 0.0013 | 0.0016 | 0.0016 | 0.0014 | 0.0111 | 0.0015 | 0.0011 | 0.0012 | 0.0014 | 0.0010 | 0.0007 | 0.0006 |  |  |  |  |
| Nov-21 | 0.0014 | 0.0015 | 0.0013 | 0.0013 | 0.0015 | 0.0013 | 0.0094 | 0.0015 | 0.0011 | 0.0011 | 0.0011 | 0.0005 | 0.0002 |  |  |  |  |  |
| Dec-21 | 0.0016 | 0.0013 | 0.0012 | 0.0015 | 0.0015 | 0.0011 | 0.0075 | 0.0014 | 0.0011 | 0.0010 | 0.0006 | 0.0003 |  |  |  |  |  |  |
| Jan-22 | 0.0015 | 0.0015 | 0.0015 | 0.0016 | 0.0016 | 0.0011 | 0.0063 | 0.0015 | 0.0014 | 0.0007 | 0.0004 | 0.0001 |  |  |  |  |  |  |
| Feb-22 | 0.0021 | 0.0020 | 0.0023 | 0.0021 | 0.0020 | 0.0016 | 0.0113 | 0.0017 | 0.0012 | 0.0008 | 0.0003 |  |  |  |  |  |  |  |
| May-22 | 0.0017 | 0.0013 | 0.0017 | 0.0018 | 0.0018 | 0.0015 | 0.0047 |  |  |  |  |  |  |  |  |  |  |  |
| Ave Post/ Ave Pre (excluding –30 to +30) | 0.66975 |  |  |  |  |  |  |  |  |  |  |  |  |  |  |  |  |  |
| Ave Post / Ave Pre (including –30 to +30) | 0.01696 |  |  |  |  |  |  |  |  |  |  |  |  |  |  |  |  |  |

Proportion of all persons with a new type 2 diabetes mellitus diagnosis who received that ICD code from 6 months prior to 360 days after SARS-CoV-2 infection, per month of the pandemic.

**Supplementary Table 2: Number of new type 2 diabetes mellitus cases by month of SARS-CoV-2 infection. These data are also represented in Figure 2.**

| **Total count** | Month of Infection | -180  to  -50 | -149  to  -120 | -119  to  -90 | -89  to  -60 | -59  to  -30 | -29  to  -8 | -7  to  +30 | 31  to  60 | 61  to  90 | 91  to  120 | 121  to  150 | 151  to  180 | 180  to  210 | 211  to  240 | 241  to  270 | 271  to  300 | 301  to  330 | 331  to  360 |
| --- | --- | --- | --- | --- | --- | --- | --- | --- | --- | --- | --- | --- | --- | --- | --- | --- | --- | --- | --- |
| 21988 | Mar-20 | 83 | 61 | 48 | 52 | 60 | 56 | 579 | 38 | 29 | 32 | 34 | 34 | 29 | 31 | 33 | 25 | <20 | 37 |
| 63018 | Apr-20 | 239 | 166 | 163 | 207 | 168 | 113 | 1421 | 111 | 95 | 76 | 87 | 98 | 91 | 82 | 70 | 87 | 83 | 80 |
| 49149 | May-20 | 163 | 118 | 163 | 128 | 77 | 67 | 754 | 92 | 71 | 76 | 77 | 92 | 73 | 52 | 62 | 71 | 68 | 57 |
| 41683 | Jun-20 | 88 | 110 | 103 | 78 | 72 | 73 | 586 | 82 | 55 | 65 | 70 | 59 | 40 | 48 | 46 | 49 | 53 | 50 |
| 66264 | Jul-20 | 160 | 118 | 105 | 116 | 88 | 84 | 760 | 112 | 95 | 92 | 56 | 74 | 79 | 70 | 82 | 74 | 58 | 44 |
| 59857 | Aug-20 | 113 | 73 | 106 | 105 | 128 | 114 | 741 | 115 | 101 | 77 | 60 | 71 | 85 | 74 | 63 | 51 | 62 | 63 |
| 55507 | Sep-20 | 65 | 79 | 89 | 126 | 95 | 98 | 611 | 98 | 71 | 55 | 53 | 68 | 51 | 56 | 72 | 61 | 59 | 48 |
| 106177 | Oct-20 | 138 | 180 | 162 | 170 | 184 | 162 | 1048 | 181 | 131 | 148 | 129 | 140 | 129 | 107 | 118 | 96 | 101 | 85 |
| 244876 | Nov-20 | 409 | 378 | 390 | 378 | 420 | 328 | 2048 | 380 | 345 | 324 | 304 | 285 | 255 | 230 | 245 | 241 | 244 | 270 |
| 253434 | Dec-20 | 468 | 434 | 446 | 516 | 597 | 354 | 2518 | 498 | 427 | 335 | 342 | 300 | 278 | 260 | 256 | 251 | 264 | 303 |
| 177844 | Jan-21 | 378 | 353 | 386 | 425 | 315 | 220 | 2013 | 392 | 270 | 240 | 193 | 215 | 219 | 170 | 178 | 205 | 198 | 178 |
| 72152 | Feb-21 | 161 | 144 | 176 | 116 | 134 | 106 | 1062 | 159 | 113 | 110 | 107 | 94 | 81 | 60 | 71 | 83 | 71 | 65 |
| 59866 | Mar-21 | 132 | 137 | 93 | 88 | 123 | 115 | 828 | 116 | 93 | 73 | 64 | 62 | 56 | 69 | 64 | 59 | 53 | 43 |
| 63475 | Apr-21 | 115 | 99 | 100 | 96 | 137 | 100 | 783 | 109 | 76 | 74 | 61 | 58 | 61 | 46 | 51 | 37 | 69 | 70 |
| 37838 | May-21 | 65 | 67 | 79 | 79 | 69 | 70 | 576 | 74 | 54 | 48 | 45 | 37 | 47 | 40 | 36 | 33 | 40 | 25 |
| 19748 | Jun-21 | 35 | 39 | 47 | 35 | 42 | 48 | 280 | 29 | <20 | <20 | 30 | <20 | 23 | <20 | 26 | 24 | <20 | <20 |
| 39539 | Jul-21 | 79 | 68 | 59 | 74 | 66 | 42 | 481 | 50 | 40 | 53 | 36 | 25 | 38 | 23 | 26 | 24 | <20 | <20 |
| 126287 | Aug-21 | 197 | 219 | 183 | 205 | 170 | 142 | 1325 | 142 | 131 | 133 | 127 | 117 | 128 | 131 | 59 | 37 | <20 | <20 |
| 120129 | Sep-21 | 192 | 191 | 175 | 187 | 194 | 130 | 1304 | 198 | 154 | 129 | 119 | 117 | 155 | 87 | 43 | <20 | <20 | <20 |
| 75960 | Oct-21 | 139 | 110 | 99 | 118 | 118 | 105 | 845 | 117 | 85 | 89 | 109 | 78 | 51 | 42 | <20 | <20 | <20 | <20 |
| 97887 | Nov-21 | 135 | 144 | 129 | 132 | 142 | 131 | 920 | 149 | 107 | 111 | 108 | 48 | 24 | <20 | <20 | <20 | <20 | <20 |
| 224781 | Dec-21 | 350 | 298 | 277 | 342 | 330 | 248 | 1681 | 321 | 249 | 218 | 126 | 64 | <20 | <20 | <20 | <20 | <20 | <20 |
| 424738 | Jan-22 | 649 | 616 | 639 | 679 | 668 | 462 | 2655 | 651 | 594 | 291 | 150 | 30 | <20 | <20 | <20 | <20 | <20 | <20 |
| 76582 | Feb-22 | 157 | 151 | 177 | 162 | 154 | 125 | 864 | 132 | 91 | 58 | 21 | <20 | <20 | <20 | <20 | <20 | <20 | <20 |
| 24794 | Mar-22 | 55 | 48 | 40 | 40 | 56 | 49 | 282 | 28 | <20 | <20 | <20 | <20 | <20 | <20 | <20 | <20 | <20 | <20 |
| 35539 | Apr-22 | 56 | 67 | 50 | 62 | 62 | 50 | 190 | <20 | <20 | <20 | <20 | <20 | <20 | <20 | <20 | <20 | <20 | <20 |
| 54951 | May-22 | 92 | 69 | 92 | 99 | 101 | 85 | 256 | <20 | <20 | <20 | <20 | <20 | <20 | <20 | <20 | <20 | <20 | <20 |
| 31044 | Jun-22 | 57 | 53 | 66 | 76 | 67 | 40 | 152 | <20 | <20 | <20 | <20 | <20 | <20 | <20 | <20 | <20 | <20 | <20 |

Total count is the total number of people in the N3C Data Enclave with COVID-19 and a record of type 2 diabetes who have at least one clinical encounter in the database greater than 6 months prior to COVID-19 diagnosis. Each row is the number of new type 2 diabetes mellitus cases reported by month of COVID-19 diagnosis. Each column is the number of days from the index date. Day 1 is the first date of COVID-19 diagnosis by either lab result or problem list.

**Supplementary Table 3:** Demographic characteristics of all patients in the N3C Data Enclave with SARS-CoV-2 infection from March 2020 to June 2022 and a clinical encounter 6 months prior to infection.

| **Month** | **Person count** | **Age** | **Female** | | **Race/Ethnicity, n (%)** | | | | | | | | | |
| --- | --- | --- | --- | --- | --- | --- | --- | --- | --- | --- | --- | --- | --- | --- |
|  |  |  | **n (%)** | **Age** | **Asian/**  **Pacific Islander** | **Age** | **Black** | **Age** | **White** | **Age** | **Hispanic/Latinx** | **Age** | **Un-known** | **Age** |
| Mar-20 | 21988 | 54 | 12070 (55) | 45 | 921  (4) | 43 | 5648  (26) | 45 | 10415  (47) | 46 | 3313 (15) | 42 | 99  (0.4) | 43 |
| Apr-20 | 63018 | 54 | 37102 (59) | 49 | 2413  (4) | 48 | 15689  (25) | 48 | 28650  (45) | 50 | 13068 (21) | 45 | 107  (0.2) | 43 |
| May-20 | 49149 | 51 | 29598 (60) | 47 | 1834  (4) | 45 | 10847  (22) | 46 | 24188  (49) | 49 | 11629 (24) | 43 | 59  (0.1) | 42 |
| Jun-20 | 41683 | 48 | 25146 (60) | 49 | 1433  (3) | 48 | 7877  (19) | 48 | 23726  (57) | 51 | 9099 (22) | 46 | 139  (0.3) | 43 |
| Jul-20 | 66264 | 46 | 40103 (61) | 45 | 1742  (3) | 41 | 12912  (19) | 42 | 40829  (62) | 47 | 12298 (19) | 41 | 253  (0.4) | 40 |
| Aug-20 | 59857 | 46 | 36204 (60) | 48 | 1570  (3) | 45 | 9110  (15) | 47 | 39750  (66) | 50 | 9890 (17) | 43 | 155  (0.3) | 39 |
| Sep-20 | 55507 | 47 | 32691 (59) | 46 | 1445  (3) | 44 | 7012  (13) | 43 | 39216  (71) | 49 | 7619 (14) | 41 | 116  (0.2) | 45 |
| Oct-20 | 106177 | 49 | 62061 (58) | 46 | 2534  (2) | 44 | 11501  (11) | 42 | 79630  (75) | 48 | 12127 (11) | 41 | 136  (0.1) | 42 |
| Nov-20 | 244876 | 48 | 143786 (59) | 49 | 5927  (2) | 49 | 25786  (11) | 49 | 185952  (76) | 51 | 26086 (11) | 47 | 439  (0.2) | 47 |
| Dec-20 | 253434 | 50 | 149547 (59) | 46 | 7091  (3) | 45 | 31757  (13) | 44 | 182328  (72) | 47 | 30440 (12) | 43 | 718  (0.3) | 39 |
| Jan-21 | 177844 | 50 | 104217 (59) | 49 | 5216  (3) | 47 | 23838  (13) | 49 | 122835  (69) | 52 | 22905 (13) | 45 | 529  (0.3) | 46 |
| Feb-21 | 72152 | 50 | 41647 (58) | 50 | 2358  (3) | 44 | 10348  (14) | 48 | 47011  (65) | 54 | 9178 (13) | 46 | 107  (0.2) | 43 |
| Mar-21 | 59866 | 48 | 34893 (58) | 51 | 1940  (3) | 47 | 8930  (15) | 48 | 38545  (64) | 54 | 6820 (11) | 46 | 50  (0.1) | 43 |
| Apr-21 | 63475 | 46 | 37704 (59) | 51 | 1838  (3) | 51 | 11917  (19) | 49 | 40070  (63) | 54 | 6632 (10) | 46 | 58  (0.1) | 48 |
| May-21 | 37838 | 48 | 22835 (60) | 47 | 943  (2) | 46 | 7651  (20) | 44 | 23756  (63) | 49 | 3663 (10) | 42 | <20 | 49 |
| Jun-21 | 19748 | 51 | 11747 (59) | 46 | 580  (3) | 48 | 3019  (15) | 41 | 13247  (67) | 49 | 1770  (9) | 41 | 23  (0.1) | 42 |
| Jul-21 | 39539 | 47 | 23260 (59) | 47 | 903  (2) | 44 | 7142  (18) | 44 | 26810  (68) | 48 | 3196  (8) | 42 | 145  (0.4) | 41 |
| Aug-21 | 126287 | 47 | 74135 (59) | 47 | 2273  (2) | 47 | 19468  (15) | 47 | 91961  (73) | 48 | 9395  (7) | 46 | 247  (0.2) | 43 |
| Sep-21 | 120129 | 47 | 70806 (59) | 48 | 1910  (2) | 46 | 15700  (13) | 44 | 91260  (76) | 50 | 8214  (7) | 42 | 151  (0.1) | 43 |
| Oct-21 | 75960 | 49 | 44359 (58) | 50 | 1143  (2) | 50 | 8060  (11) | 45 | 59436  (78) | 53 | 5046  (7) | 44 | 76  (0.1) | 39 |
| Nov-21 | 97887 | 48 | 56396 (58) | 50 | 1563  (2) | 44 | 9111  (9) | 49 | 78025  (80) | 54 | 6224  (6) | 46 | 66  (0.1) | 44 |
| Dec-21 | 224781 | 45 | 134429 (60) | 52 | 5517  (2) | 51 | 41095  (18) | 53 | 149546  (67) | 56 | 19257 (9) | 49 | 538  (0.2) | 52 |
| Jan-22 | 424738 | 46 | 257183 (61) | 45 | 13960  (3) | 44 | 60657  (14) | 45 | 295523  (70) | 47 | 42728 (10) | 43 | 1323  (0.3) | 40 |
| Feb-22 | 76582 | 51 | 46117 (60) | 50 | 2220  (3) | 48 | 7408  (10) | 49 | 57584  (75) | 53 | 6842  (9) | 44 | 227  (0.3) | 47 |
| Mar-22 | 24794 | 52 | 14986 (60) | 45 | 964  (4) | 44 | 2264 (9) | 47 | 18210  (73) | 47 | 2188 (9) | 43 | 82 (0.3) | 43 |
| Apr-22 | 35539 | 52 | 21736 (61) | 47 | 1905  (5) | 45 | 3279 (9) | 47 | 26350  (74) | 49 | 2640 (7) | 44 | 194 (0.6) | 42 |
| May-22 | 54951 | 52 | 34055 (62) | 47 | 3136  (6) | 44 | 5706 (10) | 48 | 39729  (72) | 48 | 4570  (8) | 44 | 404  (0.8) | 39 |
| Jun-22 | 31044 | 52 | 19280 (62) | 52 | 1570  (5) | 49 | 3815 (12) | 55 | 21914  (71) | 54 | 3430 (11) | 51 | 155  (0.5) | 53 |

Demographic characteristics are reported for all individuals in the sample who received a new diabetes diagnosis in the six months prior to 360 days after SARS-CoV-2 infection, across each month of the pandemic.

Page Break

**Supplementary Table 4:** SNOMED Concept Identifiers used to define type 2 diabetes mellitus**.**

| Concept Name | Concept ID | Is Excluded | Include Descendants | Include Mapped |
| --- | --- | --- | --- | --- |
| Diabetes insipidus | 30968 | TRUE | TRUE | FALSE |
| Disorder of kidney due to diabetes mellitus | 192279 | FALSE | TRUE | FALSE |
| Neonatal diabetes mellitus | 193323 | TRUE | FALSE | FALSE |
| Renal disorder due to type 1 diabetes mellitus | 200687 | TRUE | FALSE | FALSE |
| Type 1 diabetes mellitus | 201254 | TRUE | FALSE | FALSE |
| Hyperosmolar coma due to type 2 diabetes mellitus | 201530 | FALSE | FALSE | FALSE |
| Hyperosmolar coma due to type 1 diabetes mellitus | 201531 | TRUE | FALSE | FALSE |
| Diabetes mellitus | 201820 | FALSE | TRUE | FALSE |
| Type 2 diabetes mellitus | 201826 | FALSE | TRUE | FALSE |
| Peripheral circulatory disorder due to type 1 diabetes mellitus | 318712 | TRUE | FALSE | FALSE |
| Peripheral vascular disorder due to diabetes mellitus | 321822 | FALSE | FALSE | FALSE |
| Disorder of nervous system due to type 2 diabetes mellitus | 376065 | FALSE | FALSE | FALSE |
| Disorder of nervous system due to type 1 diabetes mellitus | 377821 | TRUE | FALSE | FALSE |
| Disorder due to type 1 diabetes mellitus | 435216 | TRUE | FALSE | FALSE |
| Ketoacidosis due to type 1 diabetes mellitus | 439770 | TRUE | FALSE | FALSE |
| Complication due to diabetes mellitus | 442793 | FALSE | TRUE | FALSE |
| Diabetic - poor control | 443238 | FALSE | TRUE | FALSE |
| Type 1 diabetes mellitus without complication | 443412 | TRUE | FALSE | FALSE |
| Hyperosmolality due to uncontrolled type 1 diabetes mellitus | 443592 | TRUE | FALSE | FALSE |
| Diabetic ketoacidosis | 443727 | TRUE | FALSE | FALSE |
| Peripheral circulatory disorder due to type 2 diabetes mellitus | 443729 | FALSE | FALSE | FALSE |
| Disorder of nervous system due to diabetes mellitus | 443730 | FALSE | TRUE | FALSE |
| Renal disorder due to type 2 diabetes mellitus | 443731 | FALSE | FALSE | FALSE |
| Disorder due to type 2 diabetes mellitus | 443732 | FALSE | TRUE | FALSE |
| Disorder of eye due to type 2 diabetes mellitus | 443733 | FALSE | FALSE | FALSE |
| Ketoacidosis due to type 2 diabetes mellitus | 443734 | FALSE | FALSE | FALSE |
| Disorder of eye due to diabetes mellitus | 443767 | FALSE | TRUE | FALSE |
| Peripheral vascular disease due to secondary diabetes mellitus | 760977 | FALSE | FALSE | FALSE |
| Polyneuropathy due to secondary diabetes mellitus | 760979 | FALSE | FALSE | FALSE |
| Neurogenic erectile dysfunction due to diabetes mellitus type 2 | 760989 | FALSE | FALSE | FALSE |
| Hyperosmolarity due to secondary diabetes mellitus | 761049 | FALSE | FALSE | FALSE |
| Ketoacidosis due to secondary diabetes mellitus | 761050 | FALSE | FALSE | FALSE |
| Complication due to secondary diabetes mellitus | 761051 | FALSE | TRUE | FALSE |
| Neuropathic ankle ulcer due to diabetes mellitus type 2 | 761063 | FALSE | FALSE | FALSE |
| Severe nonproliferative retinopathy due to diabetes mellitus type 1 | 765373 | TRUE | FALSE | FALSE |
| Ischemic ankle ulcer due to diabetes mellitus type 2 | 765375 | FALSE | FALSE | FALSE |
| Type 2 diabetes mellitus with ketoacidosis | 1326491 | FALSE | FALSE | FALSE |
| Type 2 diabetes mellitus with ketoacidosis without coma | 1326492 | FALSE | FALSE | FALSE |
| Type 2 diabetes mellitus with ketoacidosis with coma | 1326493 | FALSE | FALSE | FALSE |
| *NO VALUE* | 1409150 | FALSE | FALSE | FALSE |
| *NO VALUE* | 1409151 | FALSE | FALSE | FALSE |
| *NO VALUE* | 1409152 | FALSE | FALSE | FALSE |
| *NO VALUE* | 1409154 | FALSE | FALSE | FALSE |
| *NO VALUE* | 1409193 | FALSE | FALSE | FALSE |
| *NO VALUE* | 1409194 | FALSE | FALSE | FALSE |
| Type 2 diabetes mellitus | 1567956 | FALSE | FALSE | FALSE |
| Type 2 diabetes mellitus with hyperosmolarity | 1567957 | FALSE | FALSE | FALSE |
| Type 2 diabetes mellitus with kidney complications | 1567958 | FALSE | FALSE | FALSE |
| Type 2 diabetes mellitus with ophthalmic complications | 1567959 | FALSE | FALSE | FALSE |
| Type 2 diabetes mellitus with unspecified diabetic retinopathy | 1567960 | FALSE | FALSE | FALSE |
| Type 2 diabetes mellitus with mild nonproliferative diabetic retinopathy | 1567961 | FALSE | FALSE | FALSE |
| Type 2 diabetes mellitus with moderate nonproliferative diabetic retinopathy | 1567962 | FALSE | FALSE | FALSE |
| Type 2 diabetes mellitus with severe nonproliferative diabetic retinopathy | 1567963 | FALSE | FALSE | FALSE |
| Type 2 diabetes mellitus with proliferative diabetic retinopathy | 1567964 | FALSE | FALSE | FALSE |
| Type 2 diabetes mellitus with neurological complications | 1567965 | FALSE | FALSE | FALSE |
| Type 2 diabetes mellitus with circulatory complications | 1567966 | FALSE | FALSE | FALSE |
| Type 2 diabetes mellitus with other specified complications | 1567967 | FALSE | FALSE | FALSE |
| Type 2 diabetes mellitus with diabetic arthropathy | 1567968 | FALSE | FALSE | FALSE |
| Type 2 diabetes mellitus with skin complications | 1567969 | FALSE | FALSE | FALSE |
| Type 2 diabetes mellitus with oral complications | 1567970 | FALSE | FALSE | FALSE |
| Type 2 diabetes mellitus with hypoglycemia | 1567971 | FALSE | FALSE | FALSE |
| History of gestational diabetes mellitus | 3087518 | TRUE | TRUE | FALSE |
| Preulcerative calluses | 3180411 | TRUE | FALSE | FALSE |
| Iatrogenic diabetes insipidus | 3181307 | TRUE | TRUE | FALSE |
| Disorder associated with diabetes mellitus | 3182725 | FALSE | TRUE | FALSE |
| Poorly controlled type I diabetes with renal complication | 3192052 | TRUE | FALSE | FALSE |
| Poorly controlled type I diabetes with neuropathy | 3192955 | TRUE | FALSE | FALSE |
| Poorly controlled type 2 diabetes | 3193274 | FALSE | TRUE | FALSE |
| Poorly controlled type 1 diabetes | 3194119 | TRUE | FALSE | FALSE |
| Poorly controlled type I diabetes with complication | 3196797 | TRUE | FALSE | FALSE |
| Poorly controlled diabetes mellitus | 3198118 | FALSE | TRUE | FALSE |
| Poorly controlled type I diabetes with circulatory disorder | 3198350 | TRUE | FALSE | FALSE |
| History of gestational diabetes mellitus | 3346930 | TRUE | TRUE | FALSE |
| Diabetes mellitus uncontrolled | 3469133 | FALSE | TRUE | FALSE |
| Leprechaunism syndrome | 4006979 | TRUE | FALSE | FALSE |
| Diabetes mellitus without complication | 4008576 | FALSE | TRUE | FALSE |
| Diabetic ketoacidosis without coma | 4009303 | FALSE | FALSE | FALSE |
| Brittle diabetes mellitus | 4019513 | FALSE | FALSE | FALSE |
| Gestational diabetes mellitus | 4024659 | TRUE | FALSE | FALSE |
| Familial central diabetes insipidus | 4029440 | TRUE | TRUE | FALSE |
| Acrorenal field defect, ectodermal dysplasia, and lipoatrophic diabetes | 4030064 | TRUE | FALSE | FALSE |
| Photomyoclonus, diabetes mellitus, deafness, nephropathy and cerebral dysfunction | 4030066 | TRUE | FALSE | FALSE |
| Muscular atrophy, ataxia, retinitis pigmentosa, and diabetes mellitus | 4034961 | TRUE | FALSE | FALSE |
| Hyperproinsulinemia | 4034962 | TRUE | FALSE | FALSE |
| Megaloblastic anemia, thiamine-responsive, with diabetes mellitus and sensorineural deafness | 4034963 | TRUE | FALSE | FALSE |
| Hyperglycemic disorder in pregnancy | 4034966 | TRUE | TRUE | FALSE |
| Drug-induced nephrogenic diabetes insipidus | 4043348 | TRUE | TRUE | FALSE |
| Insulin dependent diabetes mellitus type 1A | 4047906 | TRUE | FALSE | FALSE |
| Houssay's syndrome | 4048202 | TRUE | FALSE | FALSE |
| Glucose tolerance test indicates diabetes mellitus | 4055679 | FALSE | FALSE | FALSE |
| At risk of diabetes mellitus | 4060085 | TRUE | FALSE | FALSE |
| Cellulitis of foot due to diabetes mellitus | 4061725 | FALSE | TRUE | FALSE |
| Pre-existing type 1 diabetes mellitus | 4063042 | TRUE | FALSE | FALSE |
| Diabetes mellitus in neonate small for gestational age | 4079850 | TRUE | FALSE | FALSE |
| Disorder of soft tissue due to diabetes mellitus | 4082346 | FALSE | TRUE | FALSE |
| Hypohidrosis-diabetes insipidus syndrome | 4082360 | TRUE | TRUE | FALSE |
| Type 1 diabetes mellitus with ulcer | 4099214 | TRUE | FALSE | FALSE |
| Type 1 diabetes mellitus maturity onset | 4099215 | TRUE | FALSE | FALSE |
| Multiple complications due to type 2 diabetes mellitus | 4099216 | FALSE | FALSE | FALSE |
| Type 2 diabetes mellitus with ulcer | 4099651 | FALSE | FALSE | FALSE |
| Fibrocalculous pancreatic diabetes | 4099741 | TRUE | FALSE | FALSE |
| Insulin dependent diabetes mellitus type 1B | 4102018 | TRUE | FALSE | FALSE |
| Diabetic hand syndrome | 4114426 | FALSE | TRUE | FALSE |
| Lipodystrophy, partial, with Rieger anomaly, short stature, and insulinopenic diabetes mellitus | 4129379 | TRUE | FALSE | FALSE |
| Bird-headed dwarfism with progressive ataxia, insulin-resistant diabetes, goiter, and primary gonadal insufficiency | 4129515 | TRUE | FALSE | FALSE |
| Insulin resistance - type A | 4129524 | TRUE | FALSE | FALSE |
| Insulin resistance - type B | 4129525 | TRUE | FALSE | FALSE |
| Insulin treated type 2 diabetes mellitus | 4130162 | FALSE | FALSE | FALSE |
| Maturity onset diabetes of the young, type 2 | 4130164 | FALSE | FALSE | FALSE |
| Hypogonadism, diabetes mellitus, alopecia, mental retardation and electrocardiographic abnormalities | 4130165 | TRUE | FALSE | FALSE |
| Lipoatrophic diabetes | 4131907 | TRUE | FALSE | FALSE |
| Small vessel disease due to type 2 diabetes mellitus | 4142579 | FALSE | FALSE | FALSE |
| Small vessel disease due to type 1 diabetes mellitus | 4143689 | TRUE | FALSE | FALSE |
| Lumbosacral radiculoplexus neuropathy due to type 1 diabetes mellitus | 4143857 | TRUE | FALSE | FALSE |
| Latent autoimmune diabetes mellitus in adult | 4145827 | TRUE | FALSE | FALSE |
| Type 1 diabetes mellitus with arthropathy | 4152858 | TRUE | FALSE | FALSE |
| Diabetic foot ulcer | 4159742 | FALSE | TRUE | FALSE |
| Neurohypophyseal diabetes insipidus | 4164730 | TRUE | TRUE | FALSE |
| Maternal diabetes mellitus with hypoglycemia affecting fetus OR newborn | 4166381 | TRUE | FALSE | FALSE |
| Familial diabetes insipidus | 4171246 | TRUE | TRUE | FALSE |
| Infection of foot due to diabetes mellitus | 4171406 | FALSE | TRUE | FALSE |
| Diabetes mellitus due to insulin receptor antibodies | 4192852 | TRUE | FALSE | FALSE |
| Type 2 diabetes mellitus without complication | 4193704 | FALSE | TRUE | FALSE |
| Arthropathy due to type 2 diabetes mellitus | 4196141 | FALSE | FALSE | FALSE |
| Type 2 diabetes mellitus with neuropathic arthropathy | 4198296 | FALSE | FALSE | FALSE |
| Type 2 diabetes mellitus with peripheral angiopathy | 4200875 | FALSE | FALSE | FALSE |
| Diabetes resolved | 4201636 | FALSE | FALSE | FALSE |
| Hyperosmolar non-ketotic state due to type 2 diabetes mellitus | 4215719 | FALSE | FALSE | FALSE |
| Acquired nephrogenic diabetes insipidus | 4216968 | TRUE | TRUE | FALSE |
| Bronze diabetes | 4220821 | FALSE | FALSE | FALSE |
| Exudative maculopathy due to type 1 diabetes mellitus | 4221344 | TRUE | FALSE | FALSE |
| Cataract due to diabetes mellitus type 2 | 4221495 | FALSE | FALSE | FALSE |
| Dipsogenic diabetes insipidus | 4221532 | TRUE | TRUE | FALSE |
| Mononeuropathy due to type 2 diabetes mellitus | 4222415 | FALSE | FALSE | FALSE |
| Persistent proteinuria due to type 1 diabetes mellitus | 4222553 | TRUE | FALSE | FALSE |
| Persistent microalbuminuria due to type 1 diabetes mellitus | 4222687 | TRUE | FALSE | FALSE |
| Gangrene due to type 1 diabetes mellitus | 4223303 | TRUE | FALSE | FALSE |
| Persistent proteinuria due to type 2 diabetes mellitus | 4223739 | FALSE | FALSE | FALSE |
| Ketoacidotic coma due to type 1 diabetes mellitus | 4224254 | TRUE | FALSE | FALSE |
| Multiple complications due to type 1 diabetes mellitus | 4224709 | TRUE | FALSE | FALSE |
| Mononeuropathy due to type 1 diabetes mellitus | 4225055 | TRUE | FALSE | FALSE |
| Cataract due to diabetes mellitus type 1 | 4225656 | TRUE | FALSE | FALSE |
| Retinopathy due to type 2 diabetes mellitus | 4226121 | FALSE | FALSE | FALSE |
| Hyperosmolar coma due to diabetes mellitus | 4226238 | FALSE | TRUE | FALSE |
| Gangrene due to diabetes mellitus | 4226354 | FALSE | TRUE | FALSE |
| Retinopathy due to type 1 diabetes mellitus | 4227210 | TRUE | FALSE | FALSE |
| Hypoglycemic coma due to type 1 diabetes mellitus | 4228112 | TRUE | FALSE | FALSE |
| Ketoacidotic coma due to type 2 diabetes mellitus | 4228443 | FALSE | FALSE | FALSE |
| Type 2 diabetes mellitus in nonobese | 4230254 | FALSE | FALSE | FALSE |
| Patient on maximal tolerated therapy for diabetes | 4236285 | FALSE | FALSE | FALSE |
| Hereditary nephrogenic diabetes insipidus | 4265337 | TRUE | TRUE | FALSE |
| Idiopathic diabetes insipidus | 4297627 | TRUE | TRUE | FALSE |
| Type 2 diabetes mellitus in obese | 4304377 | FALSE | FALSE | FALSE |
| Anemia due to diabetes mellitus | 4307799 | FALSE | TRUE | FALSE |
| Acanthosis nigricans due to type 2 diabetes mellitus | 4321756 | FALSE | FALSE | FALSE |
| Family history of diabetes mellitus type 2 | 4334340 | TRUE | FALSE | FALSE |
| bismuth subgallate 300 MG Oral Tablet | 19108331 | TRUE | FALSE | FALSE |
| fluphenazine 6 MG | 19110000 | TRUE | FALSE | FALSE |
| Type 2 diabetes mellitus with hypoglycemia without coma | 35206880 | FALSE | FALSE | FALSE |
| Type 2 diabetes mellitus with unspecified complications | 35206881 | FALSE | FALSE | FALSE |
| Type 2 diabetes mellitus without complications | 35206882 | FALSE | FALSE | FALSE |
| Macular edema due to type 1 diabetes mellitus | 35626069 | TRUE | FALSE | FALSE |
| Macular edema due to type 2 diabetes mellitus | 35626070 | FALSE | FALSE | FALSE |
| Erectile dysfunction due to diabetes mellitus | 35626763 | FALSE | TRUE | FALSE |
| Cranial nerve palsy due to type 1 diabetes mellitus | 35626765 | TRUE | FALSE | FALSE |
| Ketosis-prone diabetes mellitus | 36685758 | FALSE | TRUE | FALSE |
| Skin ulcer due to type 2 diabetes mellitus | 36712670 | FALSE | FALSE | FALSE |
| Ulcer of left foot due to type 2 diabetes mellitus | 36712686 | FALSE | FALSE | FALSE |
| Ulcer of right foot due to type 2 diabetes mellitus | 36712687 | FALSE | FALSE | FALSE |
| Peripheral angiopathy due to type 1 diabetes mellitus | 36713094 | TRUE | FALSE | FALSE |
| Atypical diabetes mellitus | 36713275 | FALSE | TRUE | FALSE |
| Hypoglycemic coma due to type 2 diabetes mellitus | 36714116 | FALSE | FALSE | FALSE |
| DEND syndrome | 36715417 | TRUE | FALSE | FALSE |
| Acidosis due to type 1 diabetes mellitus | 36715571 | TRUE | FALSE | FALSE |
| Mild nonproliferative retinopathy due to type 1 diabetes mellitus | 37016179 | TRUE | FALSE | FALSE |
| Moderate nonproliferative retinopathy due to type 1 diabetes mellitus | 37016180 | TRUE | FALSE | FALSE |
| Hyperglycemia due to type 1 diabetes mellitus | 37016348 | TRUE | FALSE | FALSE |
| Hyperglycemia due to type 2 diabetes mellitus | 37016349 | FALSE | TRUE | FALSE |
| Dyslipidemia due to type 1 diabetes mellitus | 37016353 | TRUE | FALSE | FALSE |
| Autonomic neuropathy due to type 1 diabetes mellitus | 37016767 | TRUE | FALSE | FALSE |
| Autonomic neuropathy due to type 2 diabetes mellitus | 37016768 | FALSE | FALSE | FALSE |
| Gastroparesis due to type 1 diabetes mellitus | 37017429 | TRUE | FALSE | FALSE |
| Polyneuropathy due to type 1 diabetes mellitus | 37017431 | TRUE | FALSE | FALSE |
| Polyneuropathy due to type 2 diabetes mellitus | 37017432 | FALSE | TRUE | FALSE |
| Peripheral neuropathy due to type 1 diabetes mellitus | 37018566 | TRUE | FALSE | FALSE |
| Gastroparesis due to type 2 diabetes mellitus | 37018728 | FALSE | FALSE | FALSE |
| Diabetic hand syndrome due to type 2 diabetes mellitus | 37018912 | FALSE | FALSE | FALSE |
| Permanent neonatal diabetes mellitus with cerebellar agenesis syndrome | 37110041 | TRUE | FALSE | FALSE |
| Diabetic mastopathy | 37110068 | FALSE | TRUE | FALSE |
| Stimmler syndrome | 37116379 | TRUE | FALSE | FALSE |
| Type 2 diabetes mellitus with mild nonproliferative diabetic retinopathy with macular edema, right eye | 37200198 | FALSE | FALSE | FALSE |
| Type 2 diabetes mellitus with mild nonproliferative diabetic retinopathy with macular edema, left eye | 37200199 | FALSE | FALSE | FALSE |
| Type 2 diabetes mellitus with mild nonproliferative diabetic retinopathy with macular edema, bilateral | 37200200 | FALSE | FALSE | FALSE |
| Type 2 diabetes mellitus with mild nonproliferative diabetic retinopathy with macular edema, unspecified eye | 37200201 | FALSE | FALSE | FALSE |
| Type 2 diabetes mellitus with mild nonproliferative diabetic retinopathy without macular edema, right eye | 37200202 | FALSE | FALSE | FALSE |
| Type 2 diabetes mellitus with mild nonproliferative diabetic retinopathy without macular edema, left eye | 37200203 | FALSE | FALSE | FALSE |
| Type 2 diabetes mellitus with mild nonproliferative diabetic retinopathy without macular edema, bilateral | 37200204 | FALSE | FALSE | FALSE |
| Type 2 diabetes mellitus with mild nonproliferative diabetic retinopathy without macular edema, unspecified eye | 37200205 | FALSE | FALSE | FALSE |
| Type 2 diabetes mellitus with moderate nonproliferative diabetic retinopathy with macular edema, right eye | 37200206 | FALSE | FALSE | FALSE |
| Type 2 diabetes mellitus with moderate nonproliferative diabetic retinopathy with macular edema, left eye | 37200207 | FALSE | FALSE | FALSE |
| Type 2 diabetes mellitus with moderate nonproliferative diabetic retinopathy with macular edema, bilateral | 37200208 | FALSE | FALSE | FALSE |
| Type 2 diabetes mellitus with moderate nonproliferative diabetic retinopathy with macular edema, unspecified eye | 37200209 | FALSE | FALSE | FALSE |
| Type 2 diabetes mellitus with moderate nonproliferative diabetic retinopathy without macular edema, right eye | 37200210 | FALSE | FALSE | FALSE |
| Type 2 diabetes mellitus with moderate nonproliferative diabetic retinopathy without macular edema, left eye | 37200211 | FALSE | FALSE | FALSE |
| Type 2 diabetes mellitus with moderate nonproliferative diabetic retinopathy without macular edema, bilateral | 37200212 | FALSE | FALSE | FALSE |
| Type 2 diabetes mellitus with moderate nonproliferative diabetic retinopathy without macular edema, unspecified eye | 37200213 | FALSE | FALSE | FALSE |
| Type 2 diabetes mellitus with severe nonproliferative diabetic retinopathy with macular edema, right eye | 37200214 | FALSE | FALSE | FALSE |
| Type 2 diabetes mellitus with severe nonproliferative diabetic retinopathy with macular edema, left eye | 37200215 | FALSE | FALSE | FALSE |
| Type 2 diabetes mellitus with severe nonproliferative diabetic retinopathy with macular edema, bilateral | 37200216 | FALSE | FALSE | FALSE |
| Type 2 diabetes mellitus with severe nonproliferative diabetic retinopathy with macular edema, unspecified eye | 37200217 | FALSE | FALSE | FALSE |
| Type 2 diabetes mellitus with severe nonproliferative diabetic retinopathy without macular edema, right eye | 37200218 | FALSE | FALSE | FALSE |
| Type 2 diabetes mellitus with severe nonproliferative diabetic retinopathy without macular edema, left eye | 37200219 | FALSE | FALSE | FALSE |
| Type 2 diabetes mellitus with severe nonproliferative diabetic retinopathy without macular edema, bilateral | 37200220 | FALSE | FALSE | FALSE |
| Type 2 diabetes mellitus with severe nonproliferative diabetic retinopathy without macular edema, unspecified eye | 37200221 | FALSE | FALSE | FALSE |
| Type 2 diabetes mellitus with proliferative diabetic retinopathy with macular edema, right eye | 37200222 | FALSE | FALSE | FALSE |
| Type 2 diabetes mellitus with proliferative diabetic retinopathy with macular edema, left eye | 37200223 | FALSE | FALSE | FALSE |
| Type 2 diabetes mellitus with proliferative diabetic retinopathy with macular edema, bilateral | 37200224 | FALSE | FALSE | FALSE |
| Type 2 diabetes mellitus with proliferative diabetic retinopathy with macular edema, unspecified eye | 37200225 | FALSE | FALSE | FALSE |
| Type 2 diabetes mellitus with proliferative diabetic retinopathy with traction retinal detachment involving the macula | 37200226 | FALSE | FALSE | FALSE |
| Type 2 diabetes mellitus with proliferative diabetic retinopathy with traction retinal detachment involving the macula, right eye | 37200227 | FALSE | FALSE | FALSE |
| Type 2 diabetes mellitus with proliferative diabetic retinopathy with traction retinal detachment involving the macula, left eye | 37200228 | FALSE | FALSE | FALSE |
| Type 2 diabetes mellitus with proliferative diabetic retinopathy with traction retinal detachment involving the macula, bilateral | 37200229 | FALSE | FALSE | FALSE |
| Type 2 diabetes mellitus with proliferative diabetic retinopathy with traction retinal detachment involving the macula, unspecified eye | 37200230 | FALSE | FALSE | FALSE |
| Type 2 diabetes mellitus with proliferative diabetic retinopathy with traction retinal detachment not involving the macula | 37200231 | FALSE | FALSE | FALSE |
| Type 2 diabetes mellitus with proliferative diabetic retinopathy with traction retinal detachment not involving the macula, right eye | 37200232 | FALSE | FALSE | FALSE |
| Type 2 diabetes mellitus with proliferative diabetic retinopathy with traction retinal detachment not involving the macula, left eye | 37200233 | FALSE | FALSE | FALSE |
| Type 2 diabetes mellitus with proliferative diabetic retinopathy with traction retinal detachment not involving the macula, bilateral | 37200234 | FALSE | FALSE | FALSE |
| Type 2 diabetes mellitus with proliferative diabetic retinopathy with traction retinal detachment not involving the macula, unspecified eye | 37200235 | FALSE | FALSE | FALSE |
| Type 2 diabetes mellitus with proliferative diabetic retinopathy with combined traction retinal detachment and rhegmatogenous retinal detachment | 37200236 | FALSE | FALSE | FALSE |
| Type 2 diabetes mellitus with proliferative diabetic retinopathy with combined traction retinal detachment and rhegmatogenous retinal detachment, right eye | 37200237 | FALSE | FALSE | FALSE |
| Type 2 diabetes mellitus with proliferative diabetic retinopathy with combined traction retinal detachment and rhegmatogenous retinal detachment, left eye | 37200238 | FALSE | FALSE | FALSE |
| Type 2 diabetes mellitus with proliferative diabetic retinopathy with combined traction retinal detachment and rhegmatogenous retinal detachment, bilateral | 37200239 | FALSE | FALSE | FALSE |
| Type 2 diabetes mellitus with proliferative diabetic retinopathy with combined traction retinal detachment and rhegmatogenous retinal detachment, unspecified eye | 37200240 | FALSE | FALSE | FALSE |
| Type 2 diabetes mellitus with stable proliferative diabetic retinopathy | 37200241 | FALSE | FALSE | FALSE |
| Type 2 diabetes mellitus with stable proliferative diabetic retinopathy, right eye | 37200242 | FALSE | FALSE | FALSE |
| Type 2 diabetes mellitus with stable proliferative diabetic retinopathy, left eye | 37200243 | FALSE | FALSE | FALSE |
| Type 2 diabetes mellitus with stable proliferative diabetic retinopathy, bilateral | 37200244 | FALSE | FALSE | FALSE |
| Type 2 diabetes mellitus with stable proliferative diabetic retinopathy, unspecified eye | 37200245 | FALSE | FALSE | FALSE |
| Type 2 diabetes mellitus with proliferative diabetic retinopathy without macular edema, right eye | 37200246 | FALSE | FALSE | FALSE |
| Type 2 diabetes mellitus with proliferative diabetic retinopathy without macular edema, left eye | 37200247 | FALSE | FALSE | FALSE |
| Type 2 diabetes mellitus with proliferative diabetic retinopathy without macular edema, bilateral | 37200248 | FALSE | FALSE | FALSE |
| Type 2 diabetes mellitus with proliferative diabetic retinopathy without macular edema, unspecified eye | 37200249 | FALSE | FALSE | FALSE |
| Type 2 diabetes mellitus with diabetic macular edema, resolved following treatment | 37200250 | FALSE | FALSE | FALSE |
| Type 2 diabetes mellitus with diabetic macular edema, resolved following treatment, right eye | 37200251 | FALSE | FALSE | FALSE |
| Type 2 diabetes mellitus with diabetic macular edema, resolved following treatment, left eye | 37200252 | FALSE | FALSE | FALSE |
| Type 2 diabetes mellitus with diabetic macular edema, resolved following treatment, bilateral | 37200253 | FALSE | FALSE | FALSE |
| Type 2 diabetes mellitus with diabetic macular edema, resolved following treatment, unspecified eye | 37200254 | FALSE | FALSE | FALSE |
| Woodhouse Sakati syndrome | 37311329 | TRUE | FALSE | FALSE |
| Diabetic cardiomyopathy | 37312019 | FALSE | TRUE | FALSE |
| Ulcer of midfoot due to type 1 diabetes mellitus | 37312200 | TRUE | FALSE | FALSE |
| Ulcer of heel due to type 1 diabetes mellitus | 37312201 | TRUE | FALSE | FALSE |
| Neuropathic ulcer of midfoot due to type 2 diabetes mellitus | 37312202 | FALSE | FALSE | FALSE |
| Ulcer of heel due to type 2 diabetes mellitus | 37312205 | FALSE | FALSE | FALSE |
| Neuropathy due to type 1 diabetes mellitus | 37312218 | TRUE | FALSE | FALSE |
| Gingival disease co-occurrent with diabetes mellitus | 37396524 | FALSE | TRUE | FALSE |
| sulfur Topical Lotion | 40082607 | TRUE | FALSE | FALSE |
| Multiple complications due to diabetes mellitus | 40480000 | FALSE | TRUE | FALSE |
| Type II diabetes mellitus uncontrolled | 40482801 | FALSE | TRUE | FALSE |
| Brittle type 2 diabetes mellitus | 40483315 | FALSE | FALSE | FALSE |
| Type 1 diabetes mellitus uncontrolled | 40484648 | TRUE | FALSE | FALSE |
| Type 2 diabetes mellitus well controlled | 40485020 | FALSE | FALSE | FALSE |
| History of Diabetes (regardless of treatment) [PhenX] | 40769338 | FALSE | TRUE | FALSE |
| Burgwald | 41973894 | TRUE | FALSE | FALSE |
| Disorder of nerve co-occurrent and due to type 1 diabetes mellitus | 42535539 | TRUE | FALSE | FALSE |
| Hyperosmolarity due to type 1 diabetes mellitus | 42535540 | TRUE | FALSE | FALSE |
| Absence of lower limb due to diabetes mellitus | 42536400 | FALSE | TRUE | FALSE |
| Disorder of eye due to type 1 diabetes mellitus | 42538169 | TRUE | FALSE | FALSE |
| Acute complication due to diabetes mellitus | 42538715 | FALSE | TRUE | FALSE |
| History of diabetes related lower limb amputation | 42539022 | FALSE | TRUE | FALSE |
| Gestational diabetes mellitus uncontrolled | 43020791 | TRUE | FALSE | FALSE |
| History of diabetes mellitus type 2 | 43021173 | FALSE | TRUE | FALSE |
| History of gestational diabetes mellitus | 43021968 | TRUE | TRUE | FALSE |
| On subcutaneous insulin for diabetes mellitus | 43022019 | FALSE | FALSE | FALSE |
| About how old were you when you were first told you had type 2 diabetes? | 43530492 | FALSE | FALSE | FALSE |
| Nonproliferative retinopathy due to type 2 diabetes mellitus | 43530656 | FALSE | FALSE | FALSE |
| Mixed hyperlipidemia due to type 1 diabetes mellitus | 43530660 | TRUE | FALSE | FALSE |
| Proliferative retinopathy due to type 2 diabetes mellitus | 43530685 | FALSE | FALSE | FALSE |
| Peripheral neuropathy due to type 2 diabetes mellitus | 43530689 | FALSE | FALSE | FALSE |
| Foot ulcer due to type 2 diabetes mellitus | 43530690 | FALSE | FALSE | FALSE |
| Maturity onset diabetes of the young, type 1 | 43531006 | TRUE | FALSE | FALSE |
| Diabetes mellitus due to genetic defect in beta cell function | 43531011 | FALSE | TRUE | FALSE |
| Diabetes mellitus, transient neonatal 2 | 43531019 | TRUE | FALSE | FALSE |
| Diabetes mellitus, transient neonatal 3 | 43531020 | TRUE | FALSE | FALSE |
| Chronic kidney disease stage 1 due to type 2 diabetes mellitus | 43531559 | TRUE | FALSE | FALSE |
| Chronic kidney disease stage 5 due to type 2 diabetes mellitus | 43531562 | FALSE | FALSE | FALSE |
| Dyslipidemia due to type 2 diabetes mellitus | 43531564 | FALSE | FALSE | FALSE |
| Erectile dysfunction due to type 1 diabetes mellitus | 43531565 | TRUE | FALSE | FALSE |
| Chronic kidney disease due to type 2 diabetes mellitus | 43531578 | FALSE | FALSE | FALSE |
| Angina associated with type 2 diabetes mellitus | 43531588 | FALSE | FALSE | FALSE |
| Disorder due to well controlled type 2 diabetes mellitus | 43531597 | FALSE | FALSE | FALSE |
| Vitreous hemorrhage due to type 2 diabetes mellitus | 43531608 | FALSE | FALSE | FALSE |
| Dermopathy due to type 2 diabetes mellitus | 43531616 | FALSE | FALSE | FALSE |
| Permanent neonatal diabetes mellitus | 43531641 | TRUE | FALSE | FALSE |
| Diabetes mellitus due to genetic defect in insulin action | 43531642 | FALSE | TRUE | FALSE |
| Diabetes mellitus, transient neonatal 1 | 43531645 | TRUE | FALSE | FALSE |
| Mixed hyperlipidemia due to type 2 diabetes mellitus | 43531651 | FALSE | FALSE | FALSE |
| Lipoatrophic diabetes mellitus without complication | 44787902 | TRUE | FALSE | FALSE |
| Hypoglycaemic warning impaired | 44789318 | TRUE | FALSE | FALSE |
| Hypoglycaemic warning good | 44789319 | TRUE | FALSE | FALSE |
| Maternally inherited diabetes mellitus | 44792134 | TRUE | FALSE | FALSE |
| Diabetes mellitus with multiple complications | 44793113 | FALSE | TRUE | FALSE |
| Management of gestational diabetes mellitus | 44807267 | TRUE | TRUE | FALSE |
| High risk of diabetes mellitus | 44809658 | TRUE | FALSE | FALSE |
| Hypoglycaemic warning absent | 44809809 | TRUE | FALSE | FALSE |
| Erectile dysfunction due to diabetes mellitus | 44810261 | FALSE | FALSE | FALSE |
| Diabetes mellitus | 44833365 | FALSE | FALSE | FALSE |
| Type 2 diabetes mellitus with other diabetic ophthalmic complication | 45533019 | FALSE | FALSE | FALSE |
| Type 2 diabetes mellitus with diabetic amyotrophy | 45533020 | FALSE | FALSE | FALSE |
| Type 2 diabetes mellitus with diabetic peripheral angiopathy without gangrene | 45533021 | FALSE | FALSE | FALSE |
| Type 2 diabetes mellitus with diabetic peripheral angiopathy with gangrene | 45533022 | FALSE | FALSE | FALSE |
| Type 2 diabetes mellitus with periodontal disease | 45533023 | FALSE | FALSE | FALSE |
| Type 2 diabetes mellitus with moderate nonproliferative diabetic retinopathy with macular edema | 45537961 | FALSE | FALSE | FALSE |
| Type 2 diabetes mellitus with diabetic autonomic (poly)neuropathy | 45537962 | FALSE | FALSE | FALSE |
| Type 2 diabetes mellitus with hyperosmolarity without nonketotic hyperglycemic-hyperosmolar coma (NKHHC) | 45542738 | FALSE | FALSE | FALSE |
| Type 2 diabetes mellitus with diabetic mononeuropathy | 45547625 | FALSE | FALSE | FALSE |
| Type 2 diabetes mellitus with diabetic dermatitis | 45547626 | FALSE | FALSE | FALSE |
| Type 2 diabetes mellitus with other skin complications | 45547627 | FALSE | FALSE | FALSE |
| Type 2 diabetes mellitus with mild nonproliferative diabetic retinopathy with macular edema | 45552385 | FALSE | FALSE | FALSE |
| Type 2 diabetes mellitus with proliferative diabetic retinopathy without macular edema | 45552386 | FALSE | FALSE | FALSE |
| Type 2 diabetes mellitus with severe nonproliferative diabetic retinopathy with macular edema | 45557112 | FALSE | FALSE | FALSE |
| Type 2 diabetes mellitus with diabetic polyneuropathy | 45557113 | FALSE | FALSE | FALSE |
| Type 2 diabetes mellitus with hypoglycemia with coma | 45561949 | FALSE | FALSE | FALSE |
| Type 2 diabetes mellitus with other oral complications | 45566731 | FALSE | FALSE | FALSE |
| Type 2 diabetes mellitus with other circulatory complications | 45576443 | FALSE | FALSE | FALSE |
| Type 2 diabetes mellitus with unspecified diabetic retinopathy with macular edema | 45581352 | FALSE | FALSE | FALSE |
| Type 2 diabetes mellitus with unspecified diabetic retinopathy without macular edema | 45581353 | FALSE | FALSE | FALSE |
| Type 2 diabetes mellitus with mild nonproliferative diabetic retinopathy without macular edema | 45581354 | FALSE | FALSE | FALSE |
| Type 2 diabetes mellitus with foot ulcer | 45581355 | FALSE | FALSE | FALSE |
| Type 2 diabetes mellitus with hyperosmolarity with coma | 45586139 | FALSE | FALSE | FALSE |
| Type 2 diabetes mellitus with other diabetic arthropathy | 45586140 | FALSE | FALSE | FALSE |
| Type 2 diabetes mellitus with diabetic nephropathy | 45591027 | FALSE | FALSE | FALSE |
| Type 2 diabetes mellitus with moderate nonproliferative diabetic retinopathy without macular edema | 45591029 | FALSE | FALSE | FALSE |
| Type 2 diabetes mellitus with proliferative diabetic retinopathy with macular edema | 45591030 | FALSE | FALSE | FALSE |
| Type 2 diabetes mellitus with hypoglycemia without coma | 45591031 | FALSE | FALSE | FALSE |
| Type 2 diabetes mellitus with diabetic chronic kidney disease | 45595797 | FALSE | FALSE | FALSE |
| Type 2 diabetes mellitus with diabetic cataract | 45595798 | FALSE | FALSE | FALSE |
| Type 2 diabetes mellitus with other specified complication | 45595799 | FALSE | FALSE | FALSE |
| Type 2 diabetes mellitus with diabetic neuropathic arthropathy | 45600641 | FALSE | FALSE | FALSE |
| Type 2 diabetes mellitus with other skin ulcer | 45600642 | FALSE | FALSE | FALSE |
| Type 2 diabetes mellitus with other diabetic kidney complication | 45605401 | FALSE | FALSE | FALSE |
| Type 2 diabetes mellitus with severe nonproliferative diabetic retinopathy without macular edema | 45605402 | FALSE | FALSE | FALSE |
| Type 2 diabetes mellitus with diabetic neuropathy, unspecified | 45605403 | FALSE | FALSE | FALSE |
| Type 2 diabetes mellitus with other diabetic neurological complication | 45605404 | FALSE | FALSE | FALSE |
| Type 2 diabetes mellitus with hyperglycemia | 45605405 | FALSE | FALSE | FALSE |
| Ischemia of retina due to type 1 diabetes mellitus | 45757073 | TRUE | FALSE | FALSE |
| Vitreous hemorrhage due to type 1 diabetes mellitus | 45757074 | TRUE | FALSE | FALSE |
| Diabetes mellitus due to pancreatic injury | 45757077 | FALSE | TRUE | FALSE |
| Retinal edema due to type 1 diabetes mellitus | 45757266 | TRUE | FALSE | FALSE |
| Ulcer of lower limb due to type 2 diabetes mellitus | 45757277 | FALSE | FALSE | FALSE |
| Peripheral sensory neuropathy due to type 2 diabetes mellitus | 45757278 | FALSE | FALSE | FALSE |
| Hypoglycemic unawareness due to type 1 diabetes mellitus | 45757362 | TRUE | FALSE | FALSE |
| Hypoglycemia due to type 2 diabetes mellitus | 45757363 | FALSE | FALSE | FALSE |
| Hypertension concurrent and due to end stage renal disease on dialysis due to type 2 diabetes mellitus | 45757392 | FALSE | FALSE | FALSE |
| Hypertension concurrent and due to end stage renal disease on dialysis due to type 1 diabetes mellitus | 45757393 | TRUE | FALSE | FALSE |
| Hyperlipidemia due to type 1 diabetes mellitus | 45757432 | TRUE | FALSE | FALSE |
| Hypertension in chronic kidney disease stage 3 due to type 2 diabetes mellitus | 45757446 | FALSE | FALSE | FALSE |
| Hypertension in chronic kidney disease stage 2 due to type 2 diabetes mellitus | 45757447 | FALSE | FALSE | FALSE |
| Ulcer of toe due to type 2 diabetes mellitus | 45757449 | FALSE | FALSE | FALSE |
| Diabetes mellitus type 2 without retinopathy | 45757474 | FALSE | FALSE | FALSE |
| Proteinuria due to type 2 diabetes mellitus | 45757499 | FALSE | FALSE | FALSE |
| Ulcer of foot due to type 1 diabetes mellitus | 45757507 | TRUE | FALSE | FALSE |
| Type 2 diabetes mellitus controlled by diet | 45757508 | FALSE | FALSE | FALSE |
| Microalbuminuria due to type 1 diabetes mellitus | 45757535 | TRUE | FALSE | FALSE |
| Proteinuria due to type 1 diabetes mellitus | 45757604 | TRUE | FALSE | FALSE |
| Diabetes mellitus type 1 without retinopathy | 45757674 | TRUE | FALSE | FALSE |
| Nonproliferative diabetic retinopathy due to type 1 diabetes mellitus | 45763583 | TRUE | FALSE | FALSE |
| Proliferative retinopathy due to type 1 diabetes mellitus | 45763584 | TRUE | FALSE | FALSE |
| Blindness due to type 1 diabetes mellitus | 45763585 | TRUE | FALSE | FALSE |
| Type I diabetes mellitus in remission | 45766051 | TRUE | FALSE | FALSE |
| Fetal hypertrophic cardiomyopathy due to maternal diabetes mellitus | 45766963 | TRUE | FALSE | FALSE |
| Nephrotic syndrome due to type 1 diabetes mellitus | 45769829 | TRUE | FALSE | FALSE |
| Neuropathic arthropathy due to type 1 diabetes mellitus | 45769830 | TRUE | FALSE | FALSE |
| Dermopathy due to type 1 diabetes mellitus | 45769832 | TRUE | FALSE | FALSE |
| Severe malnutrition due to type 1 diabetes mellitus | 45769833 | TRUE | FALSE | FALSE |
| Osteomyelitis due to type 1 diabetes mellitus | 45769834 | TRUE | FALSE | FALSE |
| Osteomyelitis due to type 2 diabetes mellitus | 45769836 | FALSE | FALSE | FALSE |
| Chronic ulcer of skin due to type 1 diabetes mellitus | 45769837 | TRUE | FALSE | FALSE |
| Traction detachment of retina due to type 1 diabetes mellitus | 45769873 | TRUE | FALSE | FALSE |
| Hypoglycemia due to type 1 diabetes mellitus | 45769876 | TRUE | FALSE | FALSE |
| Ulcer of forefoot due to type 2 diabetes mellitus | 45769890 | FALSE | FALSE | FALSE |
| Ankle ulcer due to type 1 diabetes mellitus | 45769891 | TRUE | FALSE | FALSE |
| Ulcer of forefoot due to type 1 diabetes mellitus | 45769892 | TRUE | FALSE | FALSE |
| Cranial nerve palsy due to type 2 diabetes mellitus | 45769894 | FALSE | FALSE | FALSE |
| Chronic kidney disease stage 2 due to type 1 diabetes mellitus | 45769901 | TRUE | FALSE | FALSE |
| Chronic kidney disease stage 4 due to type 1 diabetes mellitus | 45769902 | TRUE | FALSE | FALSE |
| Chronic kidney disease stage 5 due to type 1 diabetes mellitus | 45769903 | TRUE | FALSE | FALSE |
| End stage renal disease on dialysis due to type 1 diabetes mellitus | 45769904 | TRUE | FALSE | FALSE |
| Microalbuminuria due to type 2 diabetes mellitus | 45769905 | FALSE | FALSE | FALSE |
| End stage renal disease on dialysis due to type 2 diabetes mellitus | 45769906 | FALSE | FALSE | FALSE |
| Macular edema and retinopathy due to type 2 diabetes mellitus | 45770830 | FALSE | FALSE | FALSE |
| Hyperlipidemia due to type 2 diabetes mellitus | 45770880 | FALSE | FALSE | FALSE |
| Ulcer of lower limb due to type 1 diabetes mellitus | 45770902 | TRUE | FALSE | FALSE |
| Hypertension in chronic kidney disease due to type 1 diabetes mellitus | 45771067 | TRUE | FALSE | FALSE |
| Chronic kidney disease stage 3 due to type 1 diabetes mellitus | 45771075 | TRUE | FALSE | FALSE |
| Rubeosis iridis due to type 1 diabetes mellitus | 45771533 | TRUE | FALSE | FALSE |
| Hypoglycemia unawareness due to type 2 diabetes mellitus | 45772060 | FALSE | FALSE | FALSE |
| Sensory neuropathy due to type 1 diabetes mellitus | 45773567 | TRUE | FALSE | FALSE |
| Chronic kidney disease stage 1 due to type 1 diabetes mellitus | 45773576 | TRUE | FALSE | FALSE |
| Chronic kidney disease due to type 1 diabetes mellitus | 45773688 | TRUE | FALSE | FALSE |
| Diabetes Type 2 | 45877606 | FALSE | TRUE | FALSE |
| Skin ulcer of toe due to diabetes mellitus type 1 | 46269764 | TRUE | FALSE | FALSE |
| History of small vessel disease due to diabetes mellitus | 46270562 | FALSE | TRUE | FALSE |
| Skin ulcer of toe due to diabetes mellitus type 2 | 46274058 | FALSE | FALSE | FALSE |
